# More than tears: associations between exposure to chemical agents used by law enforcement and adverse reproductive health outcomes

**DOI:** 10.3389/fepid.2023.1177874

**Published:** 2023-08-23

**Authors:** Asha Hassan, Alison Ojanen-Goldsmith, Anna K. Hing, Madeline Mahoney, Sarah Traxler, Christy M. Boraas

**Affiliations:** ^1^ Research Division, Planned Parenthood North Central States, Minneapolis, MN, United States; ^2^ Center for Antiracism Research for Health Equity, University of Minnesota School of Public Health, Minneapolis, MN, United States; ^3^ Department of Obstetrics, Gynecology, and Women’s Health, University of Minnesota Medical School, Minneapolis, MN, United States

**Keywords:** tear gases, police violence, crowd-control, reproductive health, menstrual cycle, structural racism

## Abstract

Despite routine law enforcement use of chemical agents for crowd control, the reproductive health safety profiles of these products are unknown. Moreover, limited evidence has documented a link between such exposures and adverse reproductive health outcomes including abnormal uterine bleeding and potential pregnancy disruption. This cross-sectional study examined reproductive outcomes in adults with uteri exposed to chemical agents used by law enforcement, more commonly known as “tear gas”. Participants were recruited through social media in the wake of police violence protests. Of the 1,276 participants included in analysis, 83% reported experiencing at least one of the outcomes of interest, included uterine cramping (69%), early menstrual bleeding (55%), breast tenderness (30%), and delayed menstrual bleeding (19%). Chemical agent exposure was significantly associated with higher odds of an adverse reproductive health outcome, those with 5 days or more of exposure have 2.6 times the odds (CI: 1.61, 4.22) of adverse outcomes and having a perception that one's menstruation may fluctuate according to psychosocial stressors was associated (OR = 1.94, CI: 1.36, 2.79) with a higher odds of an adverse reproductive health experience. These findings suggest a potential relationship between exposure to chemical agents and adverse reproductive health outcomes. Given the pervasive use of these chemical agents and their potential for reproductive health harm, further investigation into the safety of these products and their impacts on individual and community health is warranted urgently.

## Introduction

On May 25th, 2020, George Floyd, an unarmed Black man, was murdered by police, sparking racial justice protests across the United States and the world throughout the summer of 2020 (1). Law enforcement frequently used “less-lethal weapons” against protestors (2), an umbrella term for chemicals and munitions deployed for crowd control that cause intentional injury and, at times although rarely, death (3). Chemical agents, colloquially referred to as “tear gas” and most commonly 2-chlorobenzalmalononitrile (CS) and phenacyl chloride (CN), are one class of these weapons that have been banned in active warfare by international treaties (4) and have been linked to many short and long-term adverse health outcomes, including blindness, glaucoma, and respiratory failure (5). However, little is known about the potential effects of chemical agents on reproductive, perinatal, or infant health. Limited evidence has linked tear gas with increased miscarriage rates after exposures in Bahrain (6), Palestine (7), and Chile (8); however, due to the reactive and uncontrolled use of chemical agents in high conflict zones, conducting methodical epidemiologic studies is challenging (9).

More recently, evidence of a dose-response relationship between to self-reported tear gas exposure and menstrual irregularities was established in Portland, Oregon utilizing cross-sectional data from summer 2020 exposures (10). Toxicology studies establishing CS and CN Immediately Dangerous to Life or Health Concentrations (IDLH) were last conducted in military settings in the 1960s and did not include surveillance for reproductive health effects (11). A 1989 article on the health effects of CS and CN gas expose a dearth of safety evidence for tear gas use, and review articles published in 2015 and 2017 echo this decades-old call for comprehensive research (9, 12, 13).

Given the well-documented scholarship on the militarization of civilian police (14), including a growing number of studies showing officers are more likely to use lethal force on people who are affected by racism or discrimination broadly or “racialized” (15) and are three times more likely to use force against movements like Black Lives Matter (16), the use of chemical agents and other less-lethal weapons during civilian protests must also be examined as a pathway for racial health inequities caused by structural racism.

Research already establishes a direct relationship between structural racism through police violence and its effect on reproductive and perinatal health (17). This body of work largely focuses on *psychosocial stress* as it relates to the impact of police violence on reproductive and perinatal outcomes. Its focus on this specific mechanism of how police violence may impact health does not account for other possible pathways between police violence and reproductive outcomes, specifically emerging factors identified during the 2020 racial justice protests like chemical exposure. The impetus for this study came from anecdotal observations of reported menstrual irregularities among protestors exposed to tear gas in May 2020. We designed this study to investigate the relationship between exposure to chemical agents and reproductive health outcomes. Therefore, we hypothesize that increased exposure to chemical agents or tear gas will be associated with increased odds of adverse reproductive health outcomes.

## Materials and methods

This cross-sectional analysis is based on data from a web-based national survey to collect preliminary data on reproductive and sexual health experiences among people with uteri exposed to chemical agents or tear gas during protests. The study team created social media and email recruitment materials that were distributed broadly between August 2020 to April 2021 via Facebook, Instagram, and to Planned Parenthood North Central States (PPNCS) email listservs. Respondents were directed to a study website with more information and a link to the REDCap survey (18). Participants completed an eligibility questionnaire and were eligible if they were a person with a uterus who was over the age of 18, able to read and write in English, and experienced tear gas exposure through inhalation or dermal contact. If eligible, participants were asked to review an information sheet about the study before proceeding to the anonymous survey. The University of Minnesota Institutional Review Board approved this research.

We examined relationships between both increased exposure to chemical agents and a history of menstrual stress response with self-reported adverse reproductive health outcomes, hypothesizing positive associations for both. We developed an online survey that included questions about demographics; menstrual and reproductive health history, including underlying health conditions, typical menstrual stress responses, and reproductive hormone use, such as birth control or gender-affirming testosterone or estrogen during the time of exposure; details on lifetime and most recent exposures to chemical agents and police weapons including time frame, duration, place, activities engaged in while exposed, type of weapon, and route of exposure; and reproductive health outcomes and details following exposures, including early or late onset menses, menstrual irregularities, duration and severity of menstrual symptoms, and pregnancy outcomes and complications.

### Measures

We measured the dependent variable, *adverse reproductive health outcomes*, by asking participants, “did you experience any of the following during or after your most recent exposure to a chemical agent used by law enforcement such as tear gas/canisters, smoke bombs/grenades, or pepper spray/mace?” Respondents who answered, “I experienced unexpected or early bleeding/period/menstruation” or “I experienced late or delayed bleeding/period/menstruation” or reported “uterine cramping” or “breast tenderness” were coded as having experienced an adverse reproductive outcome. We coded those who answered, “I did not experience any unusual or unexpected disruptions to my menstrual cycle/bleeding/period after being exposed” or reported no menstrual symptoms after exposure as not having experienced an adverse reproductive outcome.

Participants reported the *number of days of exposure to chemical agents* when asked, “How many days were you most recently exposed to chemical agents used by law enforcement/ police, such as tear gas/canisters, smoke bombs/grenades, or pepper spray/mace?” Most recently is used in this question because a previous question assessed lifetime exposure. We used quartiles to create a categorical variable of exposure, given that some people reported between 1 and 152 exposures and the distribution was quite right skewed. This variable was coded as follows Q1 “1 day,” Q2 “2 days” Q3 “more than 2 days but less than 5 days” Q4 “5 days or more.” We coded demographic controls as follows: Respondents’ self-reported *age* was categorized into four groups, 18–22 years, 23–29 years, 30–39 years, and 40 years or older. *Gender identity* was self-reported and respondents could identify as 1 “Female/cis-woman,” 2 “Male/cis-man” 3 “Genderqueer or non-binary,” 4 “Transgender female,” 5 “Transgender male,” or 6 “Other.” Because our sample was limited to people with a uterus, those who identified as 2 “Male/cis-man” and 4 “Transgender female,” were not eligible in this study. Due to small cell sizes, we had to create a dichotomous *gender identity* variable with 0 “female/cis-woman: and 1 “genderqueer, transgender female, or other.” Because a majority of the sample was white and sample sizes were small across other racial groups, we grouped race into a dichotomous *racialized* variable of 1 “white” and 2 “racialized.” “Racialized” included any person who identified as Black, American Indian/Alaska Native, Asian, Native Hawaiian/Pacific Islander, Other, Hispanic/Latinx, and Middle Eastern and North African.

The 9 census divisions (19) were collapsed into 4 *regions in alignment with US Census categorization*: 1 “Northeast (Connecticut, Maine, Massachusetts, New Hampshire, Rhode Island, Vermont, New Jersey, New York, and Pennsylvania)” 2 “Midwest (Illinois, Indiana, Michigan, Ohio, Wisconsin, Iowa, Kansas, Minnesota, Missouri, Nebraska, North Dakota, and South Dakota), 3 “South (Delaware; Florida; Georgia; Maryland; North Carolina; South Carolina; Virginia; Washington, D.C., West Virginia, Alabama, Kentucky, Mississippi, Tennessee, Arkansas, Louisiana, Oklahoma, and Texas), and 4 “West (Arizona, Colorado, Idaho, Montana, Nevada, New Mexico, Utah, Wyoming, Alaska, California, Hawaii, Oregon, and Washington).

We coded *Marital status* dichotomously as 0 “Not married” which included single, divorced, separated, and widowed, and 1 “Married” which included people who reported being married or partnered. *Combined family income* was self-reported and coded into five categories: 1 “Less than $25 K,” 2 “$25 K or greater but less than $50 K,” 3 “$50 K or greater but less than $75 k,” 4 “$75 k or greater but less than $100 k,” 5 “$100 k or greater.”

We coded health behaviors and conditions as follows: Respondents self-reported their *menstrual pattern* as 1 “Regular,” 2 “Irregular,” 3 “I do not get my period,” and 4 “Other.” Those who reported they did not get their period were excluded from this analysis due to the nature of the research question and to assess the role of stress on menstrual experience. We measured *Hormone use* dichotomously, self-reported by participants. To control for other reproductive health conditions, we created a variable for *previous reproductive health diagnosis*. We asked participants if they have ever been diagnosed with any of the following conditions: polycystic ovary syndrome, premenstrual dysphoric disorder, endometriosis, ovarian insufficiency, menopausal symptoms, overactive thyroid (hyperthyroidism) or underactive thyroid (hypothyroidism), or uterine fibroids. If they checked yes for any they were coded as 1 “Previous reproductive health diagnosis;” if none were checked they were coded as 0 “No previous reproductive health diagnosis.” Lastly, to determine if they have a *menstrual stress response history,* we asked participants “In the past, have you noticed changes in your bleeding/menstrual cycle/period when you've experienced high amounts of stress?”. If they said “Yes, I bleed earlier than normal,” “Yes, I bleed later than normal,” “Yes, I bleed heavier than usual,” or “Yes, I bleed less than normal”, we coded these participants as 1 “History of menstrual stress response.” If they responded “No, I don't notice any change” or “I’m unsure if I experience changes,” we coded them as 0 “No history of menstrual stress response”.

### Analysis

Survey responses were eligible for analysis if respondents reported tear gas exposure in the United States between in 2020 or 2021 and completed the full survey without missing data. We used STATA (20) to generate descriptive statistics for the study sample including the distribution of participant characteristics with percentages, means, and standard errors. We stratified these results by those participants reporting no adverse reproductive health outcomes and those reporting adverse reproductive health outcomes to understand how different characteristics are distributed across the outcome of interest. We conducted chi-square tests to determine if a significant difference in distribution existed between groups. Next, we created three nested logistic regression models starting with a bivariate logistic model between our key predictor—number of days of exposure to chemical agents—and our dichotomous outcome of reporting or not reporting adverse reproductive health. We then performed multivariable logistic regression with sociodemographic predictors (e.g., gender identity, combined family income) added in Model 2, and health predictors (e.g., hormone use) added in Model 3. We additionally ran a fourth model to test for moderation of a history of menstrual stress response on number of days of exposure with results provided in Supplemental Materials, as no significant results were found.

## Results

We received 2,158 survey responses and included 1,276 (59%) in the analysis.

Table 1 details descriptive statistics for participant characteristics.

**Table 1:** Distribution of study characteristics of uteri-in-situ survey participants exposed to chemical agents used by law enforcement (*N* = 1,276).

Participant characteristics	All	No adverse reproductive health outcomes		Adverse reproductive health outcomes		*p*-Value
	*N* = 1,276	*N* = 216	% = 17%	*N* = 1,060	% = 83%	
Number of days of recent exposures (mean)						.000
1st Quartile (1 day)	455	104	23%	351	77%	
2nd Quartile (2 days)	232	43	19%	189	81%	
3rd Quartile (more than 2 but less than 5 days)	230	28	12%	202	88%	
4th Quartile (5 days or more)	359	41	11%	318	89%	
Age						.939
18–22	299	50	17%	249	83%	
23–29	579	102	18%	477	82%	
30–39	317	51	16%	266	84%	
40+	81	13	16%	68	84%	
Gender						.414
Female/cis-woman	983	171	17%	812	83%	
Genderqueer, transgender male, and other	293	45	15%	248	85%	
Racialized (includes MENA & Latinx)						.777
White	983	168	17%	815	83%	
Racialized	293	48	16%	245	84%	
Region of exposure						.052
Northeast	60	17	28%	43	72%	
Midwest	315	59	19%	256	81%	
South	134	19	14%	115	86%	
West	767	121	16%	646	84%	
Marital status						.718
Not married	671	116	17%	555	83%	
Married/partnered	605	100	17%	505	83%	
Combined family income						.126
<$25 K	551	91	17%	460	83%	
>25 K but <50 k	360	53	15%	307	85%	
>50 K to <75 k	165	31	19%	134	81%	
>75 k to <100 k	88	13	15%	75	85%	
>100 k	112	28	25%	84	75%	
Menstrual pattern						.336
Regular	939	151	16%	788	84%	
Irregular	248	46	19%	202	81%	
Other	89	19	21%	70	79%	
Hormone use						.000
None	109	85	78%	24	22%	
Any	1,167	131	11%	1,036	89%	
Previous reproductive health diagnosis						.272
None	1,029	180	17%	849	83%	
Any	247	36	15%	211	85%	
Menstrual stress response history						.002
No	568	117	21%	451	79%	
Yes	708	99	14%	608	86%	

The majority of respondents (83%) reported at least one adverse reproductive health outcome following exposure to chemical agents used by law enforcement in 2020 and 2021, which included uterine cramping (69%), early menstrual bleeding (55%), breast tenderness (30%), and delayed menstrual bleeding (19%). Respondents were exposed to chemical agents for 6.1 days on average, ranging from 1 to 152 days of exposure, with 64% reporting more than one exposure day. Those who reported adverse reproductive outcomes experienced more exposure days on average (6.3) compared to those without adverse reproductive outcomes (5.1). Of note, more participants with a history of menstrual stress response reported adverse reproductive health outcomes following tear gas exposure than those without such a stress response (*p* = .002). Additionally, those who used hormones were over-represented in the group who reported an adverse reproductive health outcome.

A majority of participants identified as female cis-women (77%) and white (77%) and most reports of exposure came from the Western region of the United States (60%), followed by the Midwest (25%). Nearly half of participants were ages 23–29 years (45%) and nearly half had an annual family income of <$25 K (43%). The majority of respondents (74%) reported a regular menstrual cycle (menses once per month) although more than half (56%) reported that their cycle changed during high stress times (“menstrual stress response”). About 81% had no prior reproductive health diagnosis, and 91% were using hormones (progesterone, estrogen, or testosterone) when most recently exposed.

Table 2 shows the results of the multivariable logistic regression models. *Model 1* shows that the number of days of exposure to chemical agents is associated with higher odds of an adverse reproductive health outcome, with higher odds of an adverse outcome for the third and fourth quartiles. Individuals who experienced more than 2 but less than 5 days of exposure (Q3) had 2.14 times the odds (CI: 1.36, 3.36) of an adverse outcome compared to those who were only exposed on 1 day. While those who experienced 5 or more days of exposures (Q4) had 2.3 times the odds (CI: 1.55, 3.40) of an adverse outcome compared to those who experienced only 1 day of exposure. These associations remains the same significant when demographic and socioeconomic characteristics are added in Model 2, with Q3 participants having 2.15 times the odds (CI: 1.36, 3.40) and Q4 having 2.21 times the odds (CI: 1.47, 3.33) of adverse reproductive health outcomes compared to Q1. Additionally, *Model 2* shows that participants exposed in the Southern region of the United States have significantly higher odds of an adverse reproductive health outcome (OR = 2.33, CI: 1.09, 4.98) compared to those in the Northeast.

**Table 2:** Multivariable logistic regression of number of exposures to chemical agents by law enforcement (broken into quartiles) on adverse reproductive health outcomes of uteri-in-situ survey participants, presenting adjusted odds ratios (*N* = 1,276).

Variables	Model 1	Model 2	Model 3
	OR (CI)	OR (CI)	OR (CI)
Number of days of recent exposures			
1st Quartile (1 day)	Ref	Ref	Ref
2nd Quartile (2 days)	1.30 (.88, 1.94)	1.30 (.86, 1.93)	1.47 (.91, 2.36)
3rd Quartile (more than 2 but less than 5 days)	2.14** (1.36, 3.36)	2.15** (1.36, 3.40)	2.16** (1.27, 3.69)
4th Quartile (5 days or more)	2.30*** (1.55, 3.40)	2.21*** (1.47, 3.33)	2.61*** (1.61, 4.22)
Age category (years)			
18–22	Ref	Ref	Ref
23–29		.88 (.59, 1.29)	.97 (.62, 1.51)
30–39		1.11 (.70, 1.77)	1.17 (.68, 2.00)
40+		1.18 (.58, 2.37)	1.22 (.55, 2.73)
Gender identity			
Female ciswoman	Ref	Ref	Ref
Genderqueer, transgender male, or other		1.06 (.73, 1.54)	.97 (.62, 1.50)
Racialized		.95 (.66, 1.36)	1.17 (.75, 1.81)
Region of exposure			
Northeast	Ref	Ref	Ref
Midwest		1.58 (.83, 3.01)	1.34 (.64, 2.82)
South		2.33* (1.09, 4.98)	2.79* (1.13, 6.86)
West		1.74 (.94, 3.20)	1.80 (.88, 3.68)
Combined Family Income ($)			
25 k or less	Ref	Ref	Ref
>25 k and <=50 k		1.23 (.84, 1.82)	1.27 (.81, 2.00)
>50 k and <=75 k		.87 (.54, 1.41)	1.01 (.57, 1.79)
>75 k and <=100 k		1.18 (.62, 2.28)	1.08 (.51, 2.31)
>100 k		.58* (.35,.99)	.54* (.30,.99)
Menstrual cycle			
Regular	Ref	Ref	Ref
Irregular			.90 (.58, 1.42)
Other			.51* (.28,.91)
Hormone use			37.74*** (22.22, 64.10)
Previous diagnosis			1.24 (.78, 1.99)
Menstrual stress response history			1.94*** (1.36, 2.79)
Constant	3.38*** (2.71, 4.20)	2.08* (1.04, 4.15)	.06*** (.02,.16)

* *p* < .05.

** *p* < .01.

*** *p* < .001.

In *Model 3*, the increased exposure to chemical irritants continues to be assoiciated with higher odds of adverse reproductive health outcomes, even when menstrual patterns, underlying reproductive health diagnoses, hormone use, and menstrual stress response history are added to the model. Being exposed for more than 2 days but less than 5 (Q3) and being exposed 5 days or more (Q4) are associated with 2.16 (CI: 1.27, 3.69) and 2.61 (CI: 1.61, 4.22) times the odds of adverse reproductive health outcomes compared to being exposed one day, all else equal. Thus, across all three models, the odds of experiencing an adverse reproductive health outcome increase across quartiles, with greater exposure yielding higher adverse odds.

Further, having a menstrual stress response history is associated with almost 1.94 times the odds of reporting an adverse reproductive outcome post-exposure to tear gas (CI: 1.36, 2.79) compared to those with no menstrual stress response history. Most strikingly, those who use sex steroid hormones have over 37 times the odds of reporting an adverse reproductive health outcome in this sample (OR = 37.74, CI: 22.22, 64.10).

Lastly, to determine if a history of menstrual stress response moderated the impact of number exposures on adverse reproductive health outcomes, we ran a fourth model to test for an interaction between days of exposure to chemical irritants (in quartiles) by menstrual stress response history. As the interaction term was not significant, we refrain from discussing them at length and results are not included in Table 2. However, results from the interaction can be found in the Supplemental Materials available online.

## Discussion

### Findings

This study presents a clear association between exposure to chemical agents used by police and reported adverse reproductive health outcomes, which included uterine cramping (69%), early menstrual bleeding (55%), breast tenderness (30%), and delayed menstrual bleeding (19%). Numbers of days of exposure to tear gas, menstrual stress response history, income, hormone use, and region of exposure were significantly associated with reproductive health outcomes related to tear gas exposure. Number of days of exposure had a significant positive associations with experiencing an adverse reproductive health outcome as participants who reported adverse reproductive health outcomes. This finding confirmed our hypothesis and, while the magnitude of each adverse reproductive health outcome was not quantified in this analysis, the significant association and increased days of exposure may indicate a crude dose-response pattern which has been observed for other health outcomes previously in tear gas exposure studies (10).

While only 56% of participants reported having a menstrual cycle that was historically disrupted during times of high stress, the association between a menstrual stress response history and reporting adverse reproductive health outcomes following tear gas exposure was significant and confirmed our hypothesis. To our knowledge, this study is the first to assess the role of menstrual stress response history as a potential mediating factor in the association with chemical agent exposure. Our findings suggest several possibilities. Participants with a menstrual stress response history may have experienced adverse reproductive outcomes based on the stress and trauma of police violence experienced during protesting, regardless of tear gas deployment, which is consistent with the research literature on reproductive health impacts of police violence (21). However, our results show that tear gas exposure is still associated with these adverse reproductive health events even after controlling for menstrual stress response, suggesting that the stress experienced during protesting alone cannot explain the reported impacts on reproductive health. Another possibility is that participants with underlying irregular menstrual responses during stress may be more vulnerable to the potential reproductive health effects of tear gas exposure. This possibility may also help explain our finding that having a previous reproductive health diagnosis and underlying reproductive health conditions were also significantly associated with adverse reproductive health outcomes following tear gas exposure. These findings suggest that people with reproductive health conditions may be more vulnerable to the impacts of tear gas.

The strength of the association found with hormone use (OR = 37.74, CI: 22.22, 64.10) is likely related to the large volume of total participants (91%) who reported hormone use, such as hormonal contraceptives or gender-affirming hormone treatment, at the time of most recent tear gas exposure. These treatments are often prescribed to regulate irregular menstrual cycles and/or suppress menstruation altogether. As such, we might expect exogenous hormones to be protective against potential menstrual irregularities related to tear gas exposure due to their menstrual stabilizing and suppressing mechanisms of action. We posit that our high odds ratio indicating the opposite finding is due to overrepresentation of participant hormone use in the total sample, likely related to the recruitment methods that required social media connections to Planned Parenthood and may have included Planned Parenthood patients prescribed hormones. We theorize people who have very regular menses due to hormone use are more likely to notice a change.

Geographic region emerged as significantly associated with reproductive health outcomes. Participants exposed in the Southern United States were more likely to report adverse reproductive health outcomes (2). While the majority of participants in this study were white and not directly oppressed by structural racism, they are still impacted by the intersections of structural racism and structural violence through increased police force and weaponized responses that are specifically heightened at protests for racial justice. Police violence is in and of itself is a manifestation of structural racism (22, 23).

### Biological plausibility

Several pathophysiological pathways point to biological plausibility between tear gas and menstrual disruptions, including it metabolizing into cyanide and subsequent hypoxia and adrenal and thyroid dysregulation, indicating potential endocrine disruption (24). Additionally, CS gas is known to activate pain receptors (25) that are also involved in pain related to dysmenorrhea (26, 27), endometriosis (27, 28), and other uterine conditions (29), helping to explain uterine cramping reported by participants.

### Limitations and strengths

This study had several limitations. First, sample selection and recruitment methods presented opportunities for selection bias due to exclusive digital recruitment through social media and email listservs, targeting those with online connections to Planned Parenthood and researchers and limiting generalizability. Further, response bias may have impacted our data as people who felt most impacted by tear gas exposure may have been more likely to respond to the survey and our racially homogenous sample may indicate hesitancy by people of color to participate in health care research due to long-standing histories of medical and institutional racism. As this study relied on self-report, bias could be introduced without additional data sources like medical records to confirm outcomes. Importantly, we cannot completely disentangle the impacts of the chemical exposure from the psychological factors.

The retrospective and cross-sectional design also impact study strength. Given the uncontrolled context of law enforcement tear gas use, quantifying exposure and distinguishing between the types of chemical agents used is challenging. Participants differed in frequency and duration of exposures, ranging from repeat exposures over several months to spill-over exposure in their residential areas. This study was not designed to establish a direct causation between tear gas exposure and adverse reproductive health outcomes, as such a study would be unethical.

Despite these limitations, this study systematically collected nationwide data on tear gas exposure and related adverse reproductive health outcomes during racial justice protests in the United States, making significant contributions to the field. Our community-based sampling, while introducing potential bias compared to medical records, also allowed us to recruit participants who would or could not access health care, increasing our reach and response and including valuable health equity analyses in our study. Our study also collected robust data on participants’ menstrual and reproductive health and histories that proved significant in our models and allowed us to identify important factors for further investigation.

### Implications and further research

This study establishes an association between tear gas exposure and adverse reproductive health outcomes and strongly supports adding adverse reproductive health outcomes to the growing list of safety concerns about the use of tear gas on the public. The implications of these findings for reproductive, perinatal, maternal, and infant safety are particularly concerning, as this study identifies potential populations who may be more vulnerable to health impacts of tear gas, including pregnant people. Of note, our sample included 19 participants who reported being pregnant at the time of exposure to tear gas, 10 of whom reported a subsequent pregnancy loss (52%), a much higher rate than the expected miscarriage rate of 26% for all pregnancies (30). Given the small sample size, we cannot draw significant conclusions about tear gas exposure and pregnancy outcomes, however this finding raises numerous questions about the risks that tear gas may pose to pregnant people, and to short and long-term fertility.

We believe the evidence can only be strengthened by further studies on the risks of tear gas exposures, including confirming potential pathophysiological mechanisms for reproductive harm; in-depth analysis of the associations between adverse reproductive health outcomes, tear gas exposure, menstrual stress, hormone use and underlying reproductive health conditions; and surveillance studies that help to systematically identify actual chemical agents and the deployment procedures/conditions used by law enforcement to better describe exposure. While more evidence can help fill in scientific gaps, ultimately the onus of proving the safety of tear gas resides with manufacturers and end-users of these chemical weapons, including law enforcement and government agencies. In the absence of such evidence and reassurances and in the presence of evidence suggesting adverse effects, the precautionary principle proves relevant, and the indiscriminate use of tear gas by law enforcement warrants serious inquiry and reconsideration from policymakers. Meanwhile, increased transparency about and surveillance of chemical agents and conditions for use by law enforcement is called for to protect the public.

## Data Availability

The datasets presented in this article are not readily available to ensure participant privacy. Requests to access the datasets should be directed to ahassan@ppncs.org.
